# Effects of green tea catechin on the blood pressure and lipids in overweight and obese population-a meta-analysis

**DOI:** 10.1016/j.heliyon.2023.e21228

**Published:** 2023-11-07

**Authors:** Ying Wang, Hui Xia, Junhui Yu, Jing Sui, Da Pan, Shaokang Wang, Wang Liao, Ligang Yang, Guiju Sun

**Affiliations:** Key Laboratory of Environmental Medicine and Engineering of Ministry of Education, Department of Nutrition and Food Hygiene, School of Public Health, Southeast University, Nanjing 210009, PR China

**Keywords:** Green tea catechin, Blood pressure, Lipids, Overweight, Obese

## Abstract

**Background:**

Overweight and obesity as main health problems harm human beings worldwide. The number of people diagnosed with overweight and obese is gradually increasing. Green tea catechin has been reported to effectively help control body weight in overweight and obese population, and is protectively against the blood pressure and lipids in people with type 2 diabetes and metabolic syndrome.

**Methods:**

We retrieved 4 English databases (PubMed, Web of science, Cochrane, Scoups) from inception to April 20, 2023. Two reviewers independently determined eligibility, assessed the reporting quality of included studies, and extracted the data. Data were extracted from eleven studies. The results were presented with the weighted mean differences (WMDs), and the confidence intervals (CIs) was 95 %. The random-effects or fixed-effects model was applied according to the heterogeneity. The subgroup analysis was used to identify the source of heterogeneity. Publication bias was evaluated using funnel plots, Egger's test, and Begg's test.

**Results:**

Eleven randomized controlled trials (RCTs) inclusion studies were screened from 3072 literature articles, involving 613 overweight and obese patients. After combining all studies, it was found that in overweight and obese people green tea catechin could reduce waist circumference (WC) (pooled WMD = −1.37 cm, 95 % CI: −2.52 to −0.22 cm, p = 0.019), and triglyceride (TG) (pooled WMD = −0.18 mmol/L, 95 % CI: −0.35 to −0.02 mmol/L, p = 0.032), and increase high density lipoprotein cholesterol (HDL-c) (pooled WMD = 0.07 mmol/L, 95 % CI: 0.01–0.14 mmol/L, p = 0.031).

**Conclusion:**

Green tea catechin supplement effectively reduced WC and TG levels and improved HDL-c levels. However, it did not show the significant effect on the blood pressure in overweight and obese people. The present meta-analysis showed a moderate benefit of green tea catechin supplementation on lipid profiles in overweight and obese people.

## 1 Introduction

Obesity is a global problem, with obesity rates increasing year by year worldwide [1]. According to the World Health Organization, more than 1.9 billion adults aged 18 and older were overweight worldwide, and more than 650 million adults were obese. Thirty-nine percent of adults aged 18 years and older (39 % of men and 40 % of women) were overweight, and 13 % of the adult population (11 % of men and 15 % of women) were with obese. Studies have shown that obesity is a major risk factor for multiple chronic diseases, such as cardiovascular disease [2], hyperlipidemia [3], and diabetes [4]. The various diseases caused by obesity increase the burden of disease medical care. The economic impact of overweight and obesity is expected to rise to 3.29 % of the global GDP by 2060 [5]. Currently, studies were conducted in overweight and obese people, such as lifestyle change, behavioral therapy [6], medication, and surgery [7]. Dietary supplements play a role in improving overweight and obesity. Recently, the study showed [8] that green tea consumption has displayed the beneficial with reducing low density lipoprotein cholesterol (LDL-c) and total cholesterol (TC) levels. Furthermore, a reticular meta-analysis [9] showed that inulin, rich in green tea catechin, was a good choice for weight loss in overweight and obese patients.

Green tea is one of the most frequently cited beverages in the world [10], and tea has antioxidant properties and contains trace amounts of protein, carbohydrates, amino acids, lipids, vitamins, and minerals [11]. It also contains a wide range of compounds, but mainly polyphenols occupy the tea aroma and beneficial health effects. The main substance in green tea polyphenols is catechin, which is 25–35 % of the dry weight of green tea [12]. Green tea catechin includes (−)-epigallocatechin-3-gallate (EGCG), (−)-epicatechin-3-gallate (ECG), (−)-epigallocatechin (EGC) and (−)-epicatechin (EC) [10]. Among them, epigallocatechin gallate (EGCG) is the main active ingredient, with the most abundant content in catechin. It accounts for 50%–70 % of the catechins [12].

EGCG has been extensively studied and is considered as an effective nutritional supplement responsible for many health benefits, especially the antidiabetic [13], antiangiogenic and antimutagenic properties [14], including cholesterol-lowering [15], antimicrobial [16], prevention of Alzheimer's disease [17,18] and anti-aging [19] activities. A meta-analysis showed that green tea or green tea extract(GTE) supplementation had a significant effect on both systolic blood pressure (SBP) and diastolic blood pressure (DBP) when compared to the placebo in overweight and obese patients [20]. However, in another meta-analysis [21] showed that green tea catechin supplementation effect on the blood pressure was not significant after the subgroup analysis of overweight and obese patients. In animal experiments, supplementation with green tea extract for three months at specific doses reduced TC, triglycerides (TG), and low density lipoprotein cholesterol (LDL-c) levels in mice [22]. It has been shown that EGCG is achieved by reducing adipocyte differentiation and proliferation during adipogenesis. In population trials, green tea has significantly reduced plasma TC and LDL-c levels in overweight or obese people [21]. However, another study found that green tea catechin supplementation reduced triglyceride levels in obese people, with no effect on other serum lipid indicators [23].

Overall, the effects of the main active compound, EGCG, in green tea, on reducing obesity and improving metabolic status still remain controversial. Studies on whether green tea catechin can improve blood pressure and lipids in overweight and obese people vary. Moreover, no studies were conducted in overweight and obese adults with pooled analysis of green tea catechin on blood pressure and lipids so far. To elucidate the efficacy of green tea catechin in preventing blood pressure and dyslipidemia in overweight and obese adults. This study investigated the effects of green tea catechin on blood pressure and lipids in overweight and obese people by meta-analysis.

## 2 Methods

The current systematic review and meta-analysis were conducted based on the 2020 PRISMA guidelines [24]. The study protocol was submitted and approved in the international prospective register of systematic reviews database (PROSPERO) under the registration number: CRD42023417157.

### 2.1 Literature search strategy

We conducted electronic searches in the following data-bases: PubMed, Web of science, Scoups, and Cochrane libraries, from inception to October 31, 2022. Keywords searched were either "green tea catechin", "catechin" or "green tea extract" and "blood pressure" or "blood lipid". To search for the exact terms, quotes were used, and parentheses were used to search for group search terms. Advanced search is designed by using the Boolean operator (AND and OR), and by searching for all words derived from a keyword, using an asterisk. After searching all relevant RCTs, reference lists were also checked to find more relevant trials. The search was not restricted to publication time, gender, and language. We also performed a manual search of the relevant literatures to ensure that the retrieved literature was complete and accurate. All articles found were exported to EndNote X20 to read the title and abstract separately by the two reviewers (Ying Wang and Hui Xia). Two authors extracted the data separately, and the differences between the literature search and the literature collection were resolved through discussion and professional consultation.

### 2.2 Inclusion and exclusion criteria for the study

Inclusion criteria: (1) the study design was a randomized controlled design; (2) adult overweight and obese subjects ingesting catechin supplements; (3) the trial reported effects on SBP, SDP, TC, TG, LDL-c, and HDL-c; (4) the only difference between the test and control groups was the use of green tea catechin or green tea extracts; (5) control measures were either a placebo or a blank control. The exclusion criteria are as follows: (1) included trials of children or pregnant women; (2) studies of adding green tea catechin as a mixture; (3) the control group is low-dose catechin or other possible factors that may affect the results; (4) trials without details of the EGCG content of green tea catechin; and (5) trials with no accurate data.

### 2.3 Selection of studies and data extraction

The study selected RCTs, the data extraction by the two-person entry method, check and check to ensure that the data is accurate. The data of the 11 selected articles were extracted from BMI, waist circumference, blood pressure index (SBP, DBP), and blood lipid index (TC, TG, HDL-c, and LDL-c). Two reviewers (Ying Wang and Hui Xia) independently extracted the following characteristics of the 11 trials: first author, year, study area, study design, sample size (male/female), age, EGCG dose, baseline and endpoints of different blood pressure and lipid levels. After a rigorous discussion, results of the data extraction are summarized.

### 2.4 Assessment of the literature quality and the risk of bias

Two reviewers (Wang Ying and Hui Xia) evaluated the literature quality of the literature according to the improved Jadad scoring method, and rated the screened literature from four aspects (generation of random sequences, randomization hiding, blindness, loss to follow-up and withdrawal). Also, included risk of bias was independently assessed using the Cochrane Risk of bias tool, which assessed the risk of bias based on random sequence generation, allocation concealment, object and person blinding, outcome assessment blinding, incomplete outcome data, and selective reporting.

### 2.5 Statistical analyses

STATA15.0 were used to analyze the data, summarize and analyzed the results of the control group and the test group. Data presented in milligrams per deciliter were transformed to millimoles per liter by dividing by 38.66 for HDL-c, LDL-c, and TC while 88.6 for TG. We compare the results with the test group by calculating WMD. The pooled results were shown with the calculated WMD.

Two-sided of p < 0.05 was considered statistical signiﬁcance. Heterogeneity between trials was assessed by I^2^ and chi-squared statistics, either I^2^ > 50 % or a p-value <0.05 was considered as signiﬁcant heterogeneity. In addition, a ﬁxed-effects model was adopted to conduct the meta-analysis if statistical heterogeneity was present (p > 0.05 or I^2^ < 50 %); otherwise, a random-effects model was used. Subgroup analyses were performed to explore the sources of heterogeneity. In addition, subgroup analysis of the results according to gender, intervention dose, with and without other weight management, the data heterogeneity and sensitivity differences were analyzed. The leave-one-out method was used for sensitivity analyses to assess the impact of each research on the overall effect size. Funnel plots and Egger regression test were used to assess publication bias. To quantify the publication bias, the Egger's test and Begg's test were used [25]. The trim and fill approach was also used to rectify the funnel asymmetry induced by publication bias. A statistically significant difference was established by p < 0.05.

## 3 Results

### 3.1 Search results and characteristics of included studies

**Fig. 1:**
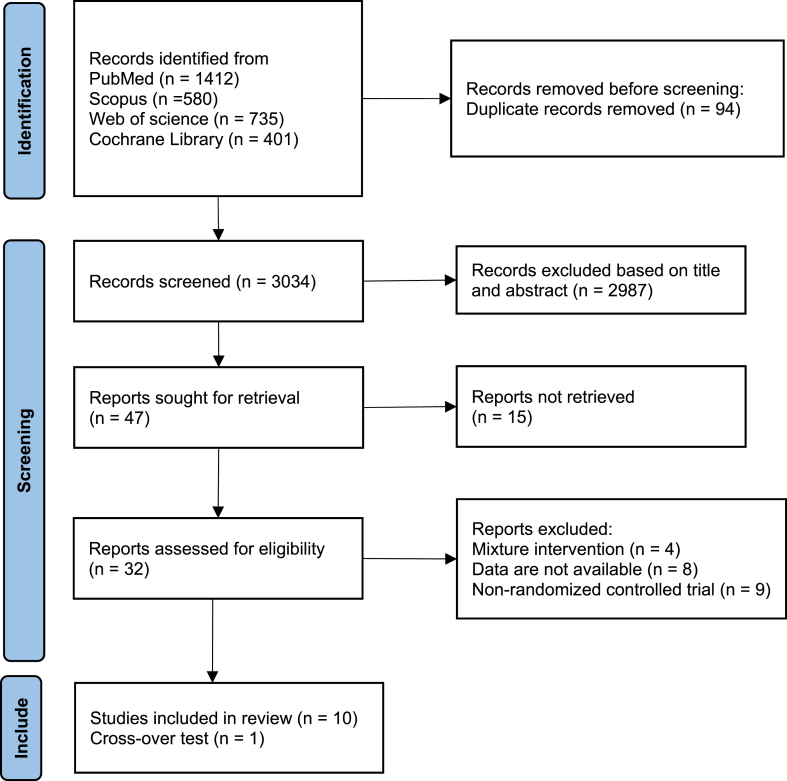
Flow diagram for selected trials. (a) Forest plot of effects of BMI (b) Forest plot of effects of waist circumference (c) Forest plot of effects of SBP (d) Forest plot of effects of DBP.

The literature search resulted in 3072 citations, 38 initially deleted for repeated publication, 2987 publications were excluded based on titles and abstracts, 15 unavailable, 4 for mixture interventions, 8 for unavailable data, 9 for non-randomized controlled trials, and 11 ultimately included in the study. A flowchart of the search strategy and study selection is shown in Fig. 1.

Table 1 summarizes the basic information of the literature, including the first author, year of publication, sample size, intervention substance, intervention dose, duration of intervention, type of placebo, etc [[26], [27], [28], [29], [30], [31], [32], [33], [34], [35], [36]].In total, the study included 613 participants, of which 313 participants were randomized to the trial group, and 300 participants were randomly assigned to the placebo-control group. In all included studies, the analysis of baseline characteristics of participants showed no significant differences between the control and trial groups in age, body weight, BMI, blood pressure, blood lipid index, and so on.

**Table 1:** Characteristics of included studies.

No.	First author	Year	Location	Number (Intervnetion/Control)	Intervention	Control	Study design	Time	Dose	Health condition	Weight management
1	I-Ju Chen [18]	2016	China	77(39/38)	Green tea Extract capsules	Cellulose	Randomized double-blind control	12 weeks	857 mg EGCG	Health	No
2	Joanna Suliburska [19]	2012	Poland	46(23/23)	Green tea Extract capsules	Cellulose	Randomized double-blind control	3 months	208 mg EGCG	Health	No
3	Juan Mielgo-Ayuso [20]	2014	Spain	83(43/40)	Green tea Extract	Lactin	Randomized double-blind control	12 weeks	300 mg EGCG	Health	Yes
4	Chung-Hua Hsu [21]	2008	China	78(41/37)	Green tea Extract capsules	Cellulose	Randomized double-blind control	12 weeks	400 mg EGCG	Health	No
5	Justin D. Roberts [22]	2021	UK	18(9/9)	Green tea Extract	Cellulose	Randomized single blind control	8 weeks	400 mg EGCG	Health	Yes
6	Chung-Hua Hsu [23]	2011	China	68(35/33)	Green tea Extract	Cellulose	Randomized double-blind control	16 weeks	857 mg EGCG	Type 2 diabetes	No
7	K. Diepvens [24]	2006	Netherlands	46(23/23)	Green tea Extract	Cellulose	Randomized double-blind control	12 weeks	267 mg EGCG	Health	Yes
8	Joanna Bajerska [25]	2015	Poland	44(23/21)	Green tea extract additive	Blank	Randomized single blind control	12 weeks	242 mg EGCG	Health	Yes
9	Tengfei Zhang [26]	2020	China	24(12/12)	Green tea Extract	Cellulose	Randomized double-blind control	12 weeks	300 mg EGCG	Health	No
10	Pawel Bogdanski [27]	2012	Poland	56(28/28)	GTE capsules	Cellulose	Randomized double-blind control	3 months	208 mg EGCG	Hypertension	No
11	Lin-Huang Huang [28]	2018	China	73(37/36)	GTE capsules	Cellulose	Randomized, double-blind, cross-over control	6 weeks	857 mg EGCG	High LDL-c	No

Table 1 shows that most of the intervention groups were green tea catechin extract capsules, one test was a green tea extract additive, and the control measure was lactose or microcrystalline cellulose. There were 2 trials with the intervention time of <12 weeks, and the rest of the intervention time was greater than or equal to 12 weeks. Five trials of the intervention dose had EGCG content greater than 300 mg, and six trials had EGCG content less than or equal to 300 mg. Furthermore, all 11 included literatures were prospective, randomized, placebo-controlled trials, with one a randomized crossover trial and one an intention-to-treat analysis trial.

### 3.2 Methodological quality and risk of bias

The quality of the literature was evaluated by JADAD scores, with a median quality score of 7 (ranging from 4 to 7). All included literature belonged to high quality literature according to the scoring rules.

The risk of bias in included literatures was assessed by the Cochrane risk bias. The risks of bias in the study are presented in Table 2. Overall, all trials (100 %) clearly described the process of randomization and how the allocation was hidden.

**Table 2:** Cochrane risk of bias assessment.

Study	Year	Random sequence generation	Allocation concealment	Blinding of participants	Blinding of outcome assessment	Free of incomplete outcome	Free of selective reporting	Other bias
Chen et al.	2016	L	L	L	L	L	L	L
Suliburska et al.	2012	U	U	L	L	L	L	L
Mielgo-Ayuso et al.	2014	L	L	L	L	L	L	L
Hsu et al.	2008	L	L	L	L	L	L	L
Roberts et al.	2021	L	L	L	L	L	L	L
Hsu et al.	2011	L	L	U	U	L	L	L
K. Diepvens et al.	2006	U	U	U	U	L	L	L
Bajerska et al.	2015	U	U	U	U	L	L	U
Zhang et al.	2020	L	L	L	L	L	L	L
Bogdanski et al.	2012	L	U	U	U	L	L	L
Huang et al.	2018	L	L	L	L	L	L	U

L, low risk of bias; H, high risk of bias; U, unclear risk of bias.

### 3.3 Summary of the anthropometric measurement results

**Fig. 2:**
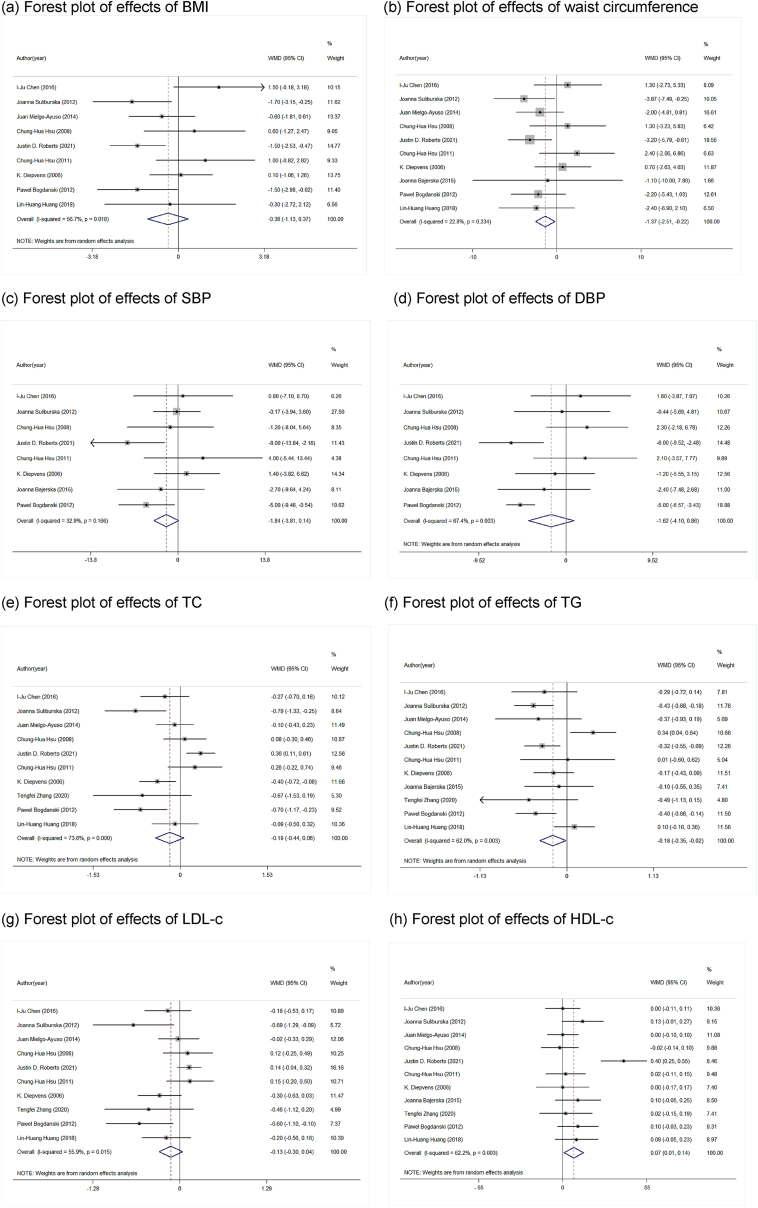
Forest plots (a) Funnel plot of effects of BMI (b) Funnel plot of effects of waist circumference.

Of eleven studies included，ten reported effects on BMI and waist circumference (WC), three trials reported effective green tea catechin supplementation in participants, and four trials reported effective green tea catechin intervention in reduced waist circumference. We summarized the results of all studies, and the results are shown in Fig. 2, including BMI (a) and WC(b). Green tea catechin supplementation had no significant effect on BMI in overweight and obese people (WMD = −0.38, 95 % CI: −1.13, 0.38, p = 0.326). Green tea catechin supplementation had a significant effect on waist circumference in overweight and obese people (WMD = −1.37,95 % CI: −2.52, −0.22, p = 0.019).

### 3.4 Summary of blood pressure results

Of eleven studies included, nine reported effects on SBP and diastolic blood pressure, two reported green tea catechin supplementation in reducing participants, and one reported it, and we summarized all the results as shown in Fig. 2, including SBP (c) and SDP (d). The results showed that green tea catechin supplementation had no significant effect on SBP (WMD = −1.84,95 % CI: −3.81, 0.14, p = 0.069) and DBP (WMD = −1.62,95 % CI: −4.10, 0.86, p = 0.201) in overweight and obese people.

### 3.5 Summary of blood lipid results

Eleven reported the effects of green tea catechin supplementation on TC, TG, LDL-c, HDL-c levels. Among them 4 reported green tea catechin supplementation effectively decreased TC levels, 3 reported green tea catechin supplementation effectively reduced TG levels, 8 reported LDL-c level, and 5 reported green tea catechin supplementation effectively increased HDL-c. However, when all data from included studies were pooled for meta-analysis, The blood lipid indexes are shown in Fig. 2, which showed that he pooled results of green tea catechin supplementation on TC (WMD = −0.19, 95 % CI: −0.44, 0.06, p = 0.142), LDL-c (WMD = −0.13, 95 % CI: −0.30, −0.04，p = 0.129), in overweight and obese people, respectively, TG (WMD = −0.18, 95 % CI: −0.35, −0.02, p = 0.032) and HDL-c (WMD = 0.07, 95 % CI: 0.01, 0.14, p = 0.031) in overweight and obese people.

### 3.6 Summary of results of the subgroup and sensitivity analysis

Subgroup analysis of all results was performed based on the study intervention dose (>300 mg vs. ≦300 mg), intervention duration (≧12 weeks vs. <12 weeks), health status (health vs. disease), weight management (yes vs. no), and the results are shown in Table 3.Detailed information on the data is summarised in Table 4.

**Table 3:** Summary results of the subgroup analysis.

Variables	Item	Standard	No. of Trials	I^2^(%)	WMD (95 % CI)	P
SBP	dose	>300 mg	4	50.3	−2.60 (−6.18,0.99)	0.213
		≦300 mg	4	27.6	−1.50 (−3.87,0.87)	0.156
	Duration	≧12weeks	7	0.0	−1.04 (−3.14,1.06)	0.332
		<12weeks	1	–	−8.00 (−13.84,-2.16)	0.007
	Health status	Health	6	27.0	−1.36 (−3.62,0.91)	0.241
		Disease	2	65.0	−3.36 (−7.39,0.68)	0.103
	Weight management	Yes	5	10.5	−1.37 (−3.80,1.06)	0.271
		No	3	63.8	−2.75 (−6.15,0.64)	0.112
DBP	dose	>300 mg	4	74.6	−0.27 (−4.92,4.38)	0.910
		≦300 mg	4	43.0	−3.05 (−5.52,-0.59)	0.015
	Duration	≧12weeks	7	68.5	−0.83 (−3.64,1.97)	0.56
		<12weeks	1	–	−6.00 (−9.52,-2.48)	0.001
	Health status	Health	6	52.6	−1.28 (−4.02,1.46)	0.359
		Disease	2	82.1	−1.99 (−8.87,4.88)	0.570
	Weight management	Yes	3	36.8	−3.51 (−6.60,-0.42)	0.026
		No	5	78.0	−0.27 (−4.21,3.67)	0.892
TC	dose	>300 mg	5	50.2	0.09 (−0.15,0.33)	0.447
		≦300 mg	5	43.6	−0.46 (−0.73,-0.19)	0.001
	Duration	≧12weeks	8	59.6	−0.28 (−0.53,-0.04)	0.024
		<12weeks	2	70.1	0.17 (−0.27,0.60)	0.458
	Health status	Health	7	76.5	−0.19 (−0.50,0.11)	0.212
		Disease	3	75.4	−0.18 (−0.70,0.35)	0.514
	Weight management	Yes	3	85.9	−0.04 (−0.50,0.42)	0.871
Variables	Item	Standard	No. of Trials	I^2^(%)	WMD (95 % CI)	P
TC	Weight management	No	7	62.8	−0.27 (−0.57,0.03)	0.078
TG	dose	>300 mg	5	72.2	−0.03 (−0.31,0.25)	0.839
		≦300 mg	6	0.0	−0.32 (−0.46,-0.19)	0.000
	Duration	≧12weeks	9	60.5	−0.20 (−0.40,-0.01)	0.044
		<12weeks	2	82.1	−0.11 (−0.53,0.30)	0.587
	Health status	Health	8	62.3	−0.21 (−0.41,-0.01)	0.044
		Disease	3	72.6	−0.12 (−0.49，0.26)	0.545
	Weight management	Yes	4	0.0	−0.24 (−0.40，-0.09)	0.002
		No	7	75.1	−0.15 (−0.42，0.11)	0.251
LDL	dose	>300 mg	5	19.0	0.041 (−0.11，0.19)	0.592
		≦300 mg	5	37.8	−0.34 (−0.59，-0.09)	0.008
	Duration	≧12weeks	8	47.9	−0.18 (−0.38，0.02)	0.071
		<12weeks	2	63.0	−0.13 (−0.30，0.04)	0.958
	Health status	Health	7	56.4	−0.12 (−0.32，0.08)	0.257
		Disease	3	66.6	−0.19 (−0.59，0.21)	0.361
	Weight management	Yes	3	63.8	−0.03 (−0.29，0.22)	0.804
		No	7	50.0	−0.20 (−0.43，0.03)	0.087
HDL	dose	>300 mg	5	82.3	0.09 (−0.05，0.23)	0.188
		≦300 mg	6	0.0	0.06 (−0.00，0.11)	0.052
	Duration	≧12weeks	9	0.0	0.03 (−0.01，0.08)	0.131
		<12weeks	2	88.6	0.24 (−0.06，0.55)	0.116
	Health status	Health	8	72.7	0.08 (−0.02，0.17)	0.107
		Disease	3	0.0	0.07 (−0.01，0.15)	0.084
	Weight management	Yes	4	85.2	0.12 (−0.06，0.31)	0.186
		No	7	0.0	0.05 (−0.01，0.10)	0.082

**Table 4:** Data summary table.

Study	Year	Mean of BMI (kg/m^2^)(CG)	SD of BMI (kg/m^2^)(CG	Mean of BMI (kg/m^2^)(IG)	SD of BMI (kg/m^2^)(IG)	Mean of WC (cm)(CG)	SD of WC (cm)(CG)	Mean of WC (cm)(IG)	SD of WC (cm)(IG)	Mean of SBP (mmHg)(CG)	SD of SBP (mmHg)(CG)	Mean of SBP(mmHg) (IG)	SD of SBP(mmHg) (IG)	Mean of DBP (mmHg)(CG)	SD of DBP (mmHg)(CG)	Mean of DBP (mmHg)(IG)	SD of DBP (mmHg)(IG)
I-Ju Chen	2016	29.1	3.6	30.6	3.9	91.5	8.2	92.8	9.8	136.2	18.5	137.0	16.8	80.6	11.1	82.2	13.3
Joanna Suliburska	2012	33.4	2.7	31.7	2.3	105.0	6.1	101.2	6.4	128.3	6.2	128.2	6.9	84.5	3.9	84.1	12.3
Juan Mielgo-Ayuso	2014	31.3	3.0	30.7	2.6	102.0	7.0	100.0	6.0	NR	NR	NR	NR	NR	NR	NR	NR
Chung-Hua Hsu	2008	30.5	4.6	31.1	3.7	91.7	11.5	93.0	8.5	132.5	16.9	131.3	13.5	79.4	10.9	81.7	9.1
Justin D. Roberts	2021	28.2	1.3	26.7	0.9	89.0	3.0	85.8	2.6	128.0	8.0	120.0	4.0	84.0	5.0	78.0	2.0
Chung-Hua Hsu	2011	29.2	3.3	30.2	4.3	94.0	8.4	96.4	10.3	142.0	19.1	146.0	20.6	85.9	10.2	88.0	13.5
K. Diepvens	2006	26.1	1.8	26.2	2.2	80.4	5.4	81.1	6.1	115.9	9.7	117.3	8.3	77.2	6.4	76.0	8.5
Joanna Bajerska	2015	NR	NR	NR	NR	104.1	15.0	103.0	15.1	134.8	15.5	132.1	5.0	86.9	8.4	84.5	8.8
Tengfei Zhang	2020	NR	NR	NR	NR	NR	NR	NR	NR	NR	NR	NR	NR	NR	NR	NR	NR
Pawel Bogdanski	2012	33.6	2.4	32.1	3.2	104.9	5.2	102.6	7.0	146.0	9.0	141.0	8.0	89.0	3.0	84.0	3.0
Lin-Huang Huang	2018	28.1	6.6	27.8	3.4	89.8	10.1	87.4	9.5	NR	NR	NR	NR	NR	NR	NR	NR

Study	Year	Mean of TC (mmol/L)(CG)	SD of TC (mmol/L)(CG)	Mean of TC (mmol/L)(IG)	SD of TC (mmol/L)(IG)	Mean of TG (mmol/L)(CG)	SD of TG (mmol/L)(CG)	Mean of TG (mmol/L)(IG)	SD of TG (mmol/L)(IG)	Mean of LDL-c (mmol/L)(CG)	SD of LDL-c (mmol/L)(CG)	Mean of LDL-c (mmol/L)<(IG)	SD of LDL-c (mmol/L)(IG)	Mean of HDL-c (mmol/L)<(CG)	SD of HDL-c (mmol/L)(CG)	Mean of HDL-c (mmol/L)	SD of HDL-c (mmol/L)(IG)
I-Ju Chen	2016	5.02	1.03	4.75	0.88	1.78	1.24	1.49	0.52	3.08	0.84	2.90	0.70	1.21	0.26	1.21	0.25
Joanna Suliburska	2012	5.65	0.92	4.86	0.94	1.66	0.46	1.23	0.41	3.71	1.12	3.02	0.96	1.15	0.21	1.28	0.26
Juan Mielgo-Ayuso	2014	4.52	0.75	4.42	0.79	2.58	1.60	2.21	0.87	2.70	0.71	2.68	0.71	1.29	0.22	1.29	0.25
Chung-Hua Hsu	2008	5.16	0.83	5.24	0.86	1.19	0.38	1.53	0.88	3.36	0.84	3.48	0.82	1.16	0.29	1.14	0.26
Justin D. Roberts	2021	5.17	0.20	5.53	0.33	1.34	0.30	1.02	0.19	3.20	0.16	3.34	0.22	1.33	0.13	1.73	0.19
Chung-Hua Hsu	2011	5.09	0.92	5.35	1.08	2.17	1.31	2.18	1.27	3.08	0.62	3.23	0.85	0.99	0.31	1.01	0.23
K. Diepvens	2006	4.90	0.50	4.50	0.60	1.10	0.50	0.93	0.40	3.00	0.50	2.70	0.60	1.40	0.30	1.40	0.30
Joanna Bajerska	2015	NR	NR	NR	NR	1.70	0.60	1.60	0.90	NR	NR	NR	NR	1.30	0.20	1.40	0.30
Tengfei Zhang	2020	5.53	1.24	4.86	0.89	1.89	0.99	1.40	0.53	3.14	0.93	2.68	0.71	1.30	0.19	1.32	0.24
Pawel Bogdanski	2012	5.70	0.90	5.00	0.90	1.50	0.50	1.10	0.50	3.70	1.00	3.10	0.90	1.30	0.20	1.40	0.30
Lin-Huang Huang	2018	5.69	1.04	5.60	0.72	1.60	0.53	1.70	0.60	3.70	0.89	3.50	0.68	1.30	0.29	1.39	0.32

Annotation: BMI:body mass index; WC: waist circumference; NR: not report; LD: low dose; HD: high dose; TC: total cholesterol; TG: total triglyceride; HDL-c: high-density lipoprotein-cholesterol; LDL-c: low-density lipoprotein-cholesterol; CG: control group; IG: intervention group.

**Fig. 3:**
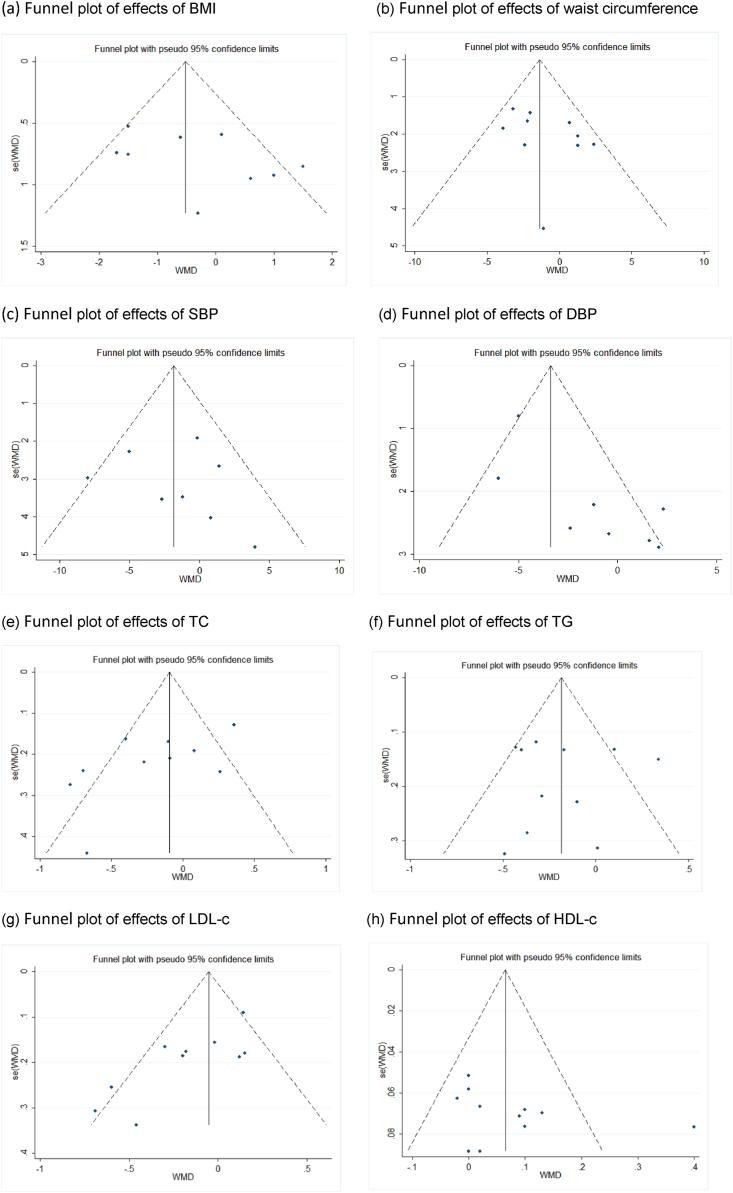
Funnel plots (a) Sensitivity analysis of effects of BMI (b) Sensitivity analysis of effects of waist circumference.

Sensitivity analyses were performed in primary outcomes by excluding studies with low quality or high risk of bias. All the meta-analyses results were not affected by the low quality or high risk of bias of studies (see Fig. 3).

### 3.7 Publication bias

**Fig. 4:**
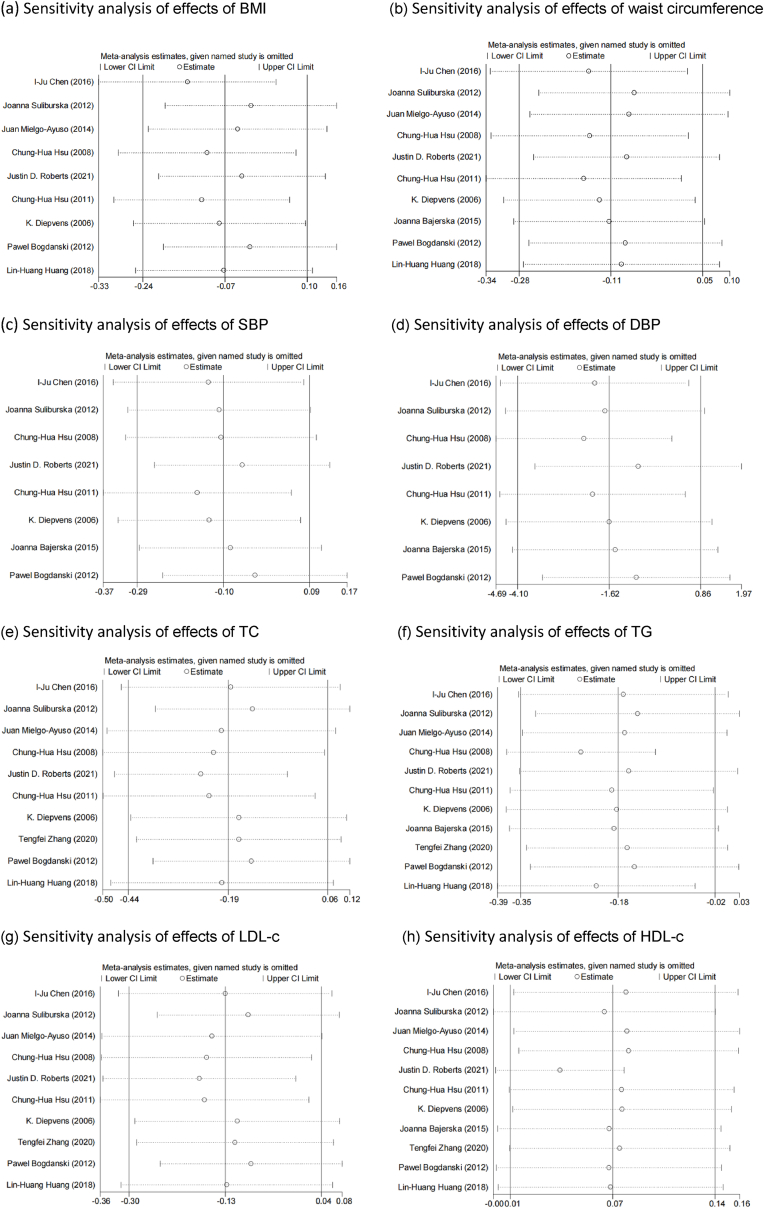
Sensitivity analysis.

According to the funnel plot (Fig. 4) and the Begg's test and Egger's tests, the results of the Begg's test were all p > 0.05 except DBP. Results with a p-value <0.05 in the Egger's test were corrected by trimming, with all corrected p-values >0.05, so that no potential publication bias was found in all the main results for BMI (Begg's test P = 0.251, Egger's test P = 0.193), WC (Begg's test P = 0.152, Egger's test P = 0.214), SBP (Begg's test P = 0.711, Egger's test P = 0.689), DBP (Begg's test P = 0.035, Egger's test P = 0.015), TC (Begg's test P = 0.283, Egger's test P = 0.061), TG (Begg's test P = 0.640, Egger's test P = 0.986), LDL-c (Begg's test P = 0.107, Egger's test P = 0.007), and HDL-c (Begg's test P = 0.161, Egger's test P = 0.247).

## 4 Discussion

This meta-analysis involved 11 RCTs with 613 subjects. This study revealed the effect of green tea catechin supplementation on the blood pressure and lipids in overweight and obese populations. We analyzed the changes in the blood pressure (SBP, DBP) and blood lipid index (TC, TG, LDL-c, and HDL-c) in the Intervention and control groups. Meanwhile, we performed a summary analysis of BMI and WC. We found that green tea catechin supplementation effectively reduced WC, TG, and increased HDL-c levels in overweight and obese populations, but had no significant effect on other indicators.

Our meta-analysis of SBP and DBP levels showed that supplementation of green tea catechins had no significant antihypertensive effect on overweight and obese people. A meta-analysis [21] showed that there was no significant change in blood pressure in subjects with BMI >30 kg/m^2^, which was consistent with our study. In animal experiments, GT was able to significantly reduce blood pressure in rats. The possible mechanism is that GT significantly activated the PI3K-Akt signaling pathway, enhanced the activity of nitric oxide synthase (eNOS) and promoted the production of nitric oxide (NO), which further exerted a blood pressure-lowering effect [37]. However, other treatments or combination therapy may be needed to reduce blood pressure in obesity patients. The possible reason is that supplementation of green tea catechins alone cannot reduce the blood pressure control of green tea catechins in overweight and obese people. It is also necessary to supplement green tea catechins combined with weight management.

There are many studies on the anti-obesity effects of green tea catechin, but the results are inconsistent. This meta-analysis found that green tea catechin supplementation had no significant effect on BMI in the overweight and obese population. A meta-analysis [38] showed that healthy participants in the treatment group lost 1.31 kg compared with the control group. However, the present meta-analysis did not show the similar results due to different population. A study by Victoria [39] showed that no significant effect of green tea on BMI (SMD = −0.22, CI −0.52 to 0.074, p = 0.14) was observed with a heterogeneity of 69.7 % (p < 0.001) whereas a significant effect of green tea on body weight (SMD = −0.7, CI −1.16 to −0.23, p = 0.003) was observed with a heterogeneity of 86.4 % (p < 0.001). At the same time, a study in Korea [40], found that catechin drink intake has no significant effect on the weight in obese women, However, combining with exercise or exercise only can effectively improve the weight of obese women, which is consistent with the present study. It showed that green tea catechin supplementation in the overweight and obese population did not show significant effect on body weight.

Interestingly, this study found that green tea catechin supplementation reduced waist circumference in overweight and obese people. The possible reason to consider is that green tea catechin is able to promote fat oxidation. Therefore, the abdominal fat and the waist circumference were reduced in overweight and obese subjects. A study [41] has found that 12 weeks of green tea catechin supplementation in overweight and obese adults has significantly reduced the total abdominal fat and the subcutaneous fat area. The reduction in abdominal fat may be due to increased abdominal fat oxidation by green tea catechin supplements. Abdominal fat oxidation may lead to a decrease in TG and free fatty acid (FFA) levels.

Studies on the mechanism of green tea catechin affecting body weight have found that green tea catechin may affect the sympathetic nervous system (SNS) [42]. SNS can increase energy consumption and promote fat oxidation, resulting in weight loss. In terms of catechin increases energy consumption, there is a hypothesis about green tea catechin can inhibit catechol-o-methyl-transferase (COMT), which is the enzyme to degrade norepinephrine (NE), thus extending the prominent gap in the sympathetic system release NE. NE is the key material to make SNS activity, make the sympathetic nervous system to increase energy consumption. The hypothesis has been explored in human trials. Studies found that separate green tea catechin failed to increase energy consumption catechin [43].Catechin and caffeine work together to increase energy expenditure [44]. The mechanism of the combination of catechin and caffeine remains further study.

The results of this study showed that supplementation of green tea catechins could effectively reduce TG levels and increase HDL-c levels in overweight and obese people. However, there was no significant effect on TC and LDL-c levels in overweight and obese people. A clinical randomized controlled trial showed that EGCG supplementation significantly reduced fasting plasma TG levels after 8 weeks of green tea catechin supplementation in obese people (p < 0.05) [16]. The results were consistent with this study, indicating that EGCG significantly reduced TG levels in overweight and obese people. There are some possible mechanisms to explain how EGCG reduces TG levels. A study in rats [45] showed that tea-catechin dose-dependently inhibited pancreatic lipase activity, thereby inhibiting triglyceride absorption and postprandial hypertriglyceridemia. In addition, green tea catechins have been shown to increase cholesterol 7α-hydroxylase gene expression in HepG2 cells [46,47] which may stimulate bile acid production and reduce cholesterol levels in hepatocytes [48]. Another study [22] on rat hepatoma cells showed that EGCG reduced the assembly and secretion of apoB-100 ultra-low density lipoprotein (VLDL) and reduced TG levels. The decrease of TG level is beneficial to the blood lipid of overweight and obese people.

More studies have found that supplementation of green tea can reduce LDL-c. A meta-analysis [49] showed that supplementation with green tea reduced TC and LDL-c levels in subjects. Another study [50] discussed the role of EGCG in reducing LDL-c. Studies have shown that EGCG in the form of beverages or capsules significantly reduces LDL-C levels in non-drug-treated healthy subjects. This is different from the results of this study. The reason for the different results may be that the research objects are different. The subjects of this study were overweight and obese people. It may be that the supplementation of green tea catechins has a more obvious effect on increasing HDL-c in overweight and obese people.

The results of subgroup analysis showed that DBP, TC, TG and LDL were significantly decreased when the dose of green tea catechin was ≦ 300 mg. Supplementation of green tea catechin time <12 weeks, SBP, DBP decreased significantly. When the time of green tea catechin supplementation ≧ 12 weeks, TC and TG indexes decreased significantly. Supplementation of green tea catechins while weight management, DBP, TG index decreased significantly. The results of subgroup analysis showed that the dose of green tea catechin should be controlled within 300 mg per day in overweight and obese people. Short-term supplementation of green tea catechins has a certain effect on blood pressure in overweight and obese people. Long-term supplementation of green tea catechins has a certain effect on blood lipid indexes of overweight and obese people. Supplementation of green tea catechins combined with weight management can effectively reduce blood pressure and blood lipid indicators.

The limitations of this study are self-evident. First, a smaller sample size in the included studies may lead to a higher risk of publication bias. This review shows that DBP has publication bias according to publication bias detection. The possible reason is that the number of research objects included in this paper is not enough. Secondly, this study did not conduct a subgroup analysis of gender because of insufficient sample size. However, in our study, due to different sex ratios, different hormone levels have different effects on obesity, blood pressure and lipids. Finally, we failed to find the exact source of heterogeneity in blood pressure and lipid parameters through subgroup analysis and meta-analysis.

## 5 Conclusion

In overweight and obese people, green tea catechin supplementation has a certain effect on lipid spectrum, which can effectively reduce the waist circumference in overweight and obese people, reduce TG and HDL-c in elevated blood lipid. However, it has no significant effect on BMI, SDP, DBP, TC, and LDL-c levels. This meta-analysis showed a moderate benefit of green tea catechin supplementation on lipid profiles in overweight and obese people, especially in reducing the function of both TG levels and elevated HDL-c levels.

## Funding statement

We appreciate that the support from the National Natural Science Foundation of China (NO. 82003457), Jiangsu Province Science Foundation for Youths (NO. BK20200366), the Fundamental Research Funds for the Central Universities and “Zhishan” Scholars Programs of Southeast University. The funders had no role in study design, data collection and analysis, decision to publish, or preparation of the manuscript.

## Author Contributions Statement

Ying Wang: Conceived and designed the experiments; Acquired of data; Analyzed and interpreted the data; Contributed reagents, materials, analysis tools or data; Wrote the paper. Hui Xia: Conceived and designed the experiments；Analyzed and interpreted the data; Revised the article. Junhui Yu and Jing Sui: Analyzed and interpreted the data Da Pan, Shaokang Wang and Wang Liao,: Contributed reagents, materials, analysis tools or data. Ligang Yang and Guiju Sun: Conceived and designed the experiments. All authors have approved the manuscript.

## Data Availability Statement

Declaration of Interest Statement All data generated or analysed during this study are included in this published article and its supplementary information files.

## Declaration of competing interest

The authors declare that they have no known competing financial interests or personal relationships that could have appeared to influence the work reported in this paper.

## References

[1] Kelly T., Yang W., Chen C.S., Reynolds K., He J. (2008). Global burden of obesity in 2005 and projections to 2030. Int. J. Obes..

[2] Powell-Wiley T.M., Poirier P., Burke L.E., Despres J.P., Gordon-Larsen P., Lavie C.J., Lear S.A., Ndumele C.E., Neeland I.J., Sanders P., St-Onge M.P., L (2021). Amer heart assoc council, N. Council cardiovasc stroke, C. Council clin, E. Council, C. Stroke, obesity and cardiovascular disease: a scientific statement from the American heart association. Circulation.

[3] Tang N., Ma J., Tao R.Q., Chen Z.J., Yang Y.D., He Q.Y., Lv Y., Lan Z.L., Zhou J.H. (2022). The effects of the interaction between BMI and dyslipidemia on hypertension in adults. Sci. Rep..

[4] Wang Q., Li N. (2020). Development of obesity and diabetes from the perspective of related guidelines and the expert consensus: a comparative study based on bibliometric analysis. Chinese Journal of Health Management.

[5] Okunogbe A., Nugent R., Spencer G., Ralston J., Wilding J. (2022). Economic impacts of overweight and obesity: current and future estimates for 161 countries. BMJ Glob. Health.

[6] Mabry-Hernandez I., Ojeda L.C. (2019). Behavioral weight loss interventions to prevent obesity-related morbidity and mortality in adults. Am. Fam. Physician.

[7] Curry S.J., Krist A.H., Owens D.K., Barry M.J., Caughey A.B., Davidson K., Doubeni C.A., Epling J.W., Grossman D.C., Kemper A.R., Kubik M., Landefeld C.S., Mangione C.M., Phipps M.G., Silverstein M., Simon M.A., Tseng C.W., Wong J.B., Force U.S.P.S.T. (2018). Behavioral weight loss interventions to prevent obesity-related morbidity and mortality in adults US preventive services task force recommendation statement. JAMA, J. Am. Med. Assoc..

[8] Xu R.F., Yang K., Li S., Dai M.Y., Chen G.Z. (2020). Effect of green tea consumption on blood lipids: a systematic review and meta-analysis of randomized controlled trials. Nutr. J..

[9] Morsali M., Poorolajal J., Shahbazi F., Vahidinia A., Doosti-Irani A. (2021). Diet therapeutics interventions for obesity: a systematic review and network meta-analysis. J. Res. Health Sci..

[10] Musial C., Kuban-Jankowska A., Gorska-Ponikowska M. (2020). Beneficial properties of green tea catechins. Int. J. Mol. Sci..

[11] Khan N., Mukhtar H. (2019). Tea polyphenols in promotion of human health. Nutrients.

[12] Senanayake S. (2013). Green tea extract: chemistry, antioxidant properties and food applications - a review. J. Funct.Foods.

[13] Zhu T.T., Li M.H., Zhu M.L., Liu X., Huang K.K., Li W.R., Wang S.X., Yin Y.L., Li P. (2022). Epigallocatechin-3-gallate alleviates type 2 diabetes mellitus via beta-cell function improvement and insulin resistance reduction. IRANIAN JOURNAL OF BASIC MEDICAL SCIENCES.

[14] da Luz J.R.D., Lopez J.A., Ferreira M.P., de Sousa R.M., Silva S.V.E., Almeida M.D., Araujo-Silva G. (2023). In vitro antithrombotic, antitumor and antiangiogenic activities of green tea polyphenols and its main constituent epigallocatechin-3-gallate. Processes.

[15] Cai J., Wang Y., Wu Y., Sun Z., Li Y., Chen X. (2017). Inhibition effects of EGCG on the Angptl3-LPL pathway in rats fed a cholesterol diet. Chin. J. Vet. Sci..

[16] Knidel C., Pereira M.F., Barcelos D.H.F., Gomes D.C.D., Guimaraes M.C.C., Schuenck R.P. (2021). Epigallocatechin gallate has antibacterial and antibiofilm activity in methicillin resistant and susceptible Staphylococcus aureus of different lineages in non-cytotoxic concentrations. Nat. Prod. Res..

[17] Lange K.W., Lange K.M., Nakamura Y. (2022). Green tea, epigallocatechin gallate and the prevention of Alzheimer?s disease: clinical evidence. Food Sci. Hum. Wellness.

[18] Youn K., Ho C.-T., Jun M. (2022). Multifaceted neuroprotective effects of (-)-epigallocatechin-3-gallate (EGCG) in Alzheimer's disease: an overview of pre-clinical studies focused on β-amyloid peptide. Food Sci. Hum. Wellness.

[19] Choi J., Kim E.M., Ko B.J., Lee U.J., Seo J.H., Kim B.G. (2022). Production of theasinensin A using laccase as antioxidant and antiaging agent. Biotechnol. Bioproc. Eng..

[20] Li G.W., Zhang Y., Thabane L., Mbuagbaw L., Liu A.P., Levine M.A.H., Holbrook A. (2015). Effect of green tea supplementation on blood pressure among overweight and obese adults: a systematic review and meta-analysis. J. Hypertens..

[21] Khalesi S., Sun J., Buys N., Jamshidi A., Nikbakht-Nasrabadi E., Khosravi-Boroujeni H. (2014). Green tea catechins and blood pressure: a systematic review and meta-analysis of randomised controlled trials. Eur. J. Nutr..

[22] Sae-Tan S., Grove K.A., Lambert J.D. (2011). Weight control and prevention of metabolic syndrome by green tea. Pharmacol. Res..

[23] Chatree S., Sitticharoon C., Maikaew P., Pongwattanapakin K., Keadkraichaiwat I., Churintaraphan M., Sripong C., Sririwichitchai R., Tapechum S. (2021). Epigallocatechin gallate decreases plasma triglyceride, blood pressure, and serum kisspeptin in obese human subjects. Exp. Biol. Med..

[24] Page M.J., McKenzie J.E., Bossuyt P.M., Boutron I., Hoffmann T.C., Mulrow C.D., Shamseer L., Tetzlaff J.M., Akl E.A., Brennan S.E., Chou R.G., Glanville J., Grimshaw J.M., Hrobjartsson A., Lalu M.M., Li T.J., Loder E.W., Mayo-Wilson E., McDonald S., McGuinness L.A., Stewart L.A., Thomas J., Tricco A.C., Welch V.A., Whiting P., Moher D. (2021). The PRISMA 2020 statement: an updated guideline for reporting systematic reviews. J. Clin. Epidemiol..

[25] Egger M., Smith G.D., Schneider M., Minder C. (1997). Bias in meta-analysis detected by a simple, graphical test. BMJ Br. Med. J. (Clin. Res. Ed.).

[26] Hsu C.H., Liao Y.L., Lin S.C., Tsai T.H., Huang C.J., Chou P. (2011). Does supplementation with green tea extract improve insulin resistance in obese type 2 diabetics? A randomized, double-blind, and placebo-controlled clinical trial. Alternative Med. Rev..

[27] Hsu C.H., Tsai T.H., Kao Y.H., Hwang K.C., Tseng T.Y., Chou P. (2008). Effect of green tea extract on obese women: a randomized, double-blind, placebo-controlled clinical trial. Clin. Nutr..

[28] Mielgo-Ayuso J., Barrenechea L., Alcorta P., Larrarte E., Margareto J., Labayen I. (2014). Effects of dietary supplementation with epigallocatechin-3-gallate on weight loss, energy homeostasis, cardiometabolic risk factors and liver function in obese women: randomised, double-blind, placebo-controlled clinical trial. Br. J. Nutr..

[29] Zhang T.F., Li N.X., Chen S., Hou Z.Q., Saito A. (2020). Effects of green tea extract combined with brisk walking on lipid profiles and the liver function in overweight and obese men: a randomized, double-blinded, placebo-control trial. An Acad. Bras Ciências.

[30] Huang L.H., Liu C.Y., Wang L.Y., Huang C.J., Hsu C.H. (2018). Effects of green tea extract on overweight and obese women with high levels of low density-lipoprotein-cholesterol (LDL-C): a randomised, double-blind, and cross-over placebo-controlled clinical trial. BMC Compl. Alternative Med..

[31] Suliburska J., Bogdanski P., Szulinska M., Stepien M., Pupek-Musialik D., Jablecka A. (2012). Effects of green tea supplementation on elements, total antioxidants, lipids, and glucose values in the serum of obese patients. Biol. Trace Elem. Res..

[32] Bajerska J., Mildner-Szkudlarz S., Walkowiak J. (2015). Effects of rye bread enriched with green tea extract on weight maintenance and the characteristics of metabolic syndrome following weight loss: a pilot study. J. Med. Food.

[33] Bogdanski P., Suliburska J., Szulinska M., Stepien M., Pupek-Musialik D., Jablecka A. (2012). Green tea extract reduces blood pressure, inflammatory biomarkers, and oxidative stress and improves parameters associated with insulin resistance in obese, hypertensive patients. Nutr. Res..

[34] Roberts J.D., Willmott A.G.B., Beasley L., Boal M., Davies R., Martin L., Chichger H., Gautam L., Del Coso J. (2021). The impact of decaffeinated green tea extract on fat oxidation, body composition and cardio-metabolic health in overweight, recreationally active individuals. Nutrients.

[35] Diepvens K., Kovacs E.M.R., Vogels N., Westerterp-Plantenga M.S. (2006). Metabolic effects of green tea and of phases of weight loss. Physiol. Behav..

[36] Chen I.J., Liu C.Y., Chiu J.P., Hsu C.H. (2016). Therapeutic effect of high-dose green tea extract on weight reduction: a randomized, double-blind, placebo-controlled clinical trial. Clin. Nutr..

[37] Wu M.R., Wu X.B., Zhu J.X., Li F.L., Wei X.L., Wang Y.F. (2022). Selenium-enriched and ordinary green tea extracts prevent high blood pressure and alter gut microbiota composition of hypertensive rats caused by high-salt diet. Food Sci. Hum. Wellness.

[38] Macedo A.P.A., Goncalves M.D.S., Barreto Medeiros J.M., David J.M., Villarreal C.F., Macambira S.G., Soares M.B.P., Couto R.D. (2022). Potential therapeutic effects of green tea on obese lipid profile - a systematic review. Nutr. health.

[39] Mahmoodi M., Hosseini R., Kazemi A., Ofori-Asenso R., Mazidi M., Mazloomi S.M. (2020). Effects of green tea or green tea catechin on liver enzymes in healthy individuals and people with nonalcoholic fatty liver disease: a systematic review and meta-analysis of randomized clinical trials. Phytother Res..

[40] ho c.w., Lee J.-W. (2012). Effect of catechins on serum lipids in obese women. The Journal of the Korea Contents Association.

[41] Maki K.C., Reeves M.S., Farmer M., Yasunaga K., Matsuo N., Katsuragi Y., Komikado M., Tokimitsu I., Wilder D., Jones F., Blumberg J.B., Cartwright Y. (2009). Green tea catechin consumption enhances exercise-induced abdominal fat loss in overweight and obese adults. J. Nutr..

[42] Rains T.M., Agarwal S., Maki K.C. (2011). Antiobesity effects of green tea catechins: a mechanistic review. J. Nutr. Biochem..

[43] Kawada T. (2014). No effect of epigallocatechin-3-gallate with weight loss on adiposity reduction, cardiometabolic risk factors and liver function in pre-menopausal obese women. Br. J. Nutr..

[44] Belza A., Toubro S., Astrup A. (2009). The effect of caffeine, green tea and tyrosine on thermogenesis and energy intake. Eur. J. Clin. Nutr..

[45] Legeay S., Rodier M., Fillon L., Faure S., Clere N. (2015). Epigallocatechin gallate: a review of its beneficial properties to prevent metabolic syndrome. Nutrients.

[46] Annaba F., Kumar P., Dudeja A.K., Saksena S., Gill R.K., Alrefai W.A. (2010). Green tea catechin EGCG inhibits ileal apical sodium bile acid transporter ASBT. Am. J. Physiol. Gastrointest. Liver Physiol..

[47] Lee M.-S., Park J.-Y., Freake H., Kwun I.-S., Kim Y. (2008). Green tea catechin enhances cholesterol 7α-hydroxylase gene expression in HepG2 cells. Br. J. Nutr..

[48] Lange K.W. (2022). Tea in cardiovascular health and disease: a critical appraisal of the evidence. Food Sci. Hum. Wellness.

[49] Yuan F., Dong H., Fang K., Gong J., Lu F.E. (2018). Effects of green tea on lipid metabolism in overweight or obese people: a meta-analysis of randomized controlled trials. Mol. Nutr. Food Res..

[50] Momose Y., Maeda-Yamamoto M., Nabetani H. (2016). Systematic review of green tea epigallocatechin gallate in reducing low-density lipoprotein cholesterol levels of humans. Int. J. Food Sci. Nutr..

