# Supplementary material for: Effects of green tea catechin on the blood pressure and lipids in overweight and obese population-a meta-analysis
Source: Heliyon. 2023 Nov 7;9(11):e21228. doi: 10.1016/j.heliyon.2023.e21228 (PMC10681946; doi:10.1016/j.heliyon.2023.e21228)
Supplement: Multimedia component 1 [file mmc1.docx]

**Supplementary Table S1.**Description of population, intervention, comparison and outcome (PICO).

| **Population** | Overweight and obese adults( ≥18 years) |
| --- | --- |
| **Intervention** | Green tea catechin supplementation |
| **Comparison** | Blank control or placebo control |
| **Outcome** | Blood pressure and blood lipid indexes |

**Supplementary Table S2.**Search strategies including the key terms and the queries for each

database.

| **Database 2022/10/31** | **Key terms and the queries** |
| --- | --- |
| Pubmed  (n=1412) | #1 "catechin"[MeSH Terms] OR "catechin"[All Fields] OR "catechins"[All Fields] OR "catechine"[All Fields] OR "catechines"[All Fields] OR "tea"[MeSH Terms] OR "tea"[All Fields] OR ("green"[All Fields] AND "tea"[All Fields]) OR "green tea"[All Fields] OR "extract"[All Fields] OR "extract's"[All Fields] OR "extractabilities"[All Fields] OR "extractability"[All Fields] OR "extractable"[All Fields] OR "extractables"[All Fields] OR "extractant"[All Fields] OR "extractants"[All Fields] OR "extracted"[All Fields] OR "extractibility"[All Fields] OR "extractible"[All Fields] OR "extracting"[All Fields] OR "extraction"[All Fields] OR "extractions"[All Fields] OR "extractive"[All Fields] OR "extractives"[All Fields] OR "extracts"[All Fields]  #2 "blood pressure"[MeSH Terms] OR ("blood"[All Fields] AND "pressure"[All Fields]) OR "blood pressure"[All Fields] OR "blood pressure determination"[MeSH Terms] OR ("blood"[All Fields] AND "pressure"[All Fields] AND "determination"[All Fields]) OR "blood pressure determination"[All Fields] OR ("blood"[All Fields] AND "pressure"[All Fields]) OR "blood pressure"[All Fields] OR "arterial pressure"[MeSH Terms] OR ("arterial"[All Fields] AND "pressure"[All Fields]) OR "arterial pressure"[All Fields] OR ("blood"[All Fields] AND "pressure"[All Fields])  #3 #1 AND #2 |
| Web of science  (n=735) | #1 TOPIC: ("catechin*") OR TOPIC: ("green tea extract*") OR TOPIC: ("green tea catechin*") OR TOPIC: ("catechine*")  #2 TOPIC: ("blood pressure") OR TOPIC: ("blood lipid") OR TOPIC: ("blood fat")  #3 #1 AND #2 |
| Cochrane  (n=401) | MeSH descriptor: [Catechin] explode all trees |
| Scopus  (n=580) | #1 TITLE-ABS-KEY ("green tea catechin*") OR TITLE-ABS-KEY ("catechin*") OR TITLE-ABS-KEY ("catechine*") OR TITLE-ABS-KEY ("green tea extract*")  #2 TITLE-ABS-KEY ("blood pressure") OR TITLE-ABS-KEY ( "arterial pressure" ) OR TITLE-ABS-KEY ( "blood lipid" ) OR TITLE-ABS-KEY ( "blood fat" )  #3 #1 AND #2 |

**Supplementary Table S3.** Inclusion and exclusion criteria for the study.

| Inclusion criteria | (1) the study design was a randomized controlled design |
| --- | --- |
|  | (2) adult overweight and obese subjects ingesting catechin supplements |
|  | (3) the trial reported effects on SBP, SDP , TC, TG, LDL-c, and HDL-c |
|  | (4) the only difference between the test and control groups was the use of green tea catechin or green tea extracts |
|  | (5) control measures were either a placebo or a blank control |
| exclusion criteria | (1) included trials of children or pregnant women |
|  | (2) studies of adding green tea catechin as a mixture |
|  | (3) the control group is low-dose catechin or other possible factors that may affect the results |
|  | (4) trials without details of the EGCG content of green tea catechin |
|  | (5) trials with no accurate data |
